# Bis(2-methyl­anilinium) diaqua­bis[dihydrogendiphosphato(2−)]cobaltate(II)

**DOI:** 10.1107/S1600536809044079

**Published:** 2009-10-31

**Authors:** Ahmed Selmi, Samah Akriche, Mohamed Rzaigui

**Affiliations:** a Laboratoire de Chimie des Matériaux, Faculté des Sciences de Bizerte, 7021 Zarzouna Bizerte, Tunisia

## Abstract

In the title cobalt(II) complex with 2-methyl­anilinium and diphosphate, (C_7_H_10_N)_2_[Co(H_2_P_2_O_7_)_2_(H_2_O)_2_], a three-dimensional network is built up from anionic layers of [Co(H_2_P_2_O_7_)_2_(H_2_O)_2_]^2−^ units and 2-methyl­anilinium cations located between these layers. The dihydrogendiphosphate groups present a bent eclipsed conformation, while the Co^2+^ ions lie on inversion centers. An intricate network of O—H⋯O and N—H⋯O hydrogen bonds is established between the different components, assuring the cohesion of the network with other inter­actions, being of electrostatic and van der Waals nature.

## Related literature

For organic-inorganic transition metal frameworks, see: Cheetham *et al.* (1999 ▶); Clearfield (1998 ▶). For the role played by diphosphates in inter­actions between metal centers, see: Xu *et al.* (2008 ▶). For related structures, see: Essehli *et al.* (2006 ▶); Gharbi *et al.* (1994 ▶); Gharbi & Jouini (2004 ▶).
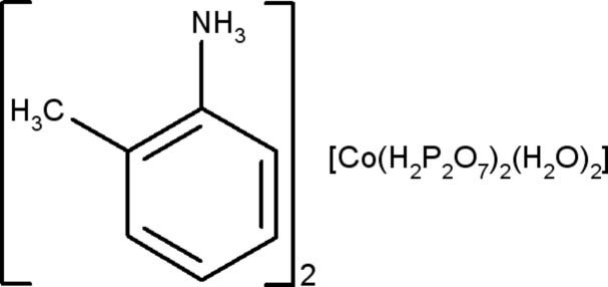


## Experimental

### 

#### Crystal data

(C_7_H_10_N)_2_[Co(H_2_P_2_O_7_)_2_(H_2_O)_2_]
*M* *_r_* = 663.19
Triclinic, 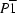

*a* = 7.440 (4) Å
*b* = 7.455 (2) Å
*c* = 11.747 (3) Å
α = 91.92 (3)°
β = 94.09 (5)°
γ = 104.67 (2)°
*V* = 627.8 (4) Å^3^
*Z* = 1
Ag *K*α radiation
μ = 0.53 mm^−1^
*T* = 298 K
0.33 × 0.26 × 0.23 mm

#### Data collection

Enraf–Nonius CAD-4 diffractometer
Absorption correction: none
4678 measured reflections
4486 independent reflections
3911 reflections with *I* > 2σ(*I*)
*R*_int_ = 0.008
2 standard reflections frequency: 120 min intensity decay: 7%

#### Refinement

*R*[*F* ^2^ > 2σ(*F* ^2^)] = 0.028
*wR*(*F* ^2^) = 0.078
*S* = 1.08
4486 reflections
181 parameters
3 restraints
H atoms treated by a mixture of independent and constrained refinement
Δρ_max_ = 0.43 e Å^−3^
Δρ_min_ = −0.29 e Å^−3^

### 

Data collection: *CAD-4 EXPRESS* (Enraf–Nonius, 1994 ▶); cell refinement: *CAD-4 EXPRESS*; data reduction: *XCAD4* (Harms & Wocadlo, 1996 ▶); program(s) used to solve structure: *SHELXS86* (Sheldrick, 2008 ▶); program(s) used to refine structure: *SHELXL97* (Sheldrick, 2008 ▶); molecular graphics: *ORTEP-3 for Windows* (Farrugia, 1997 ▶) and *DIAMOND* (Brandenburg & Putz, 2005 ▶); software used to prepare material for publication: *WinGX* (Farrugia, 1999 ▶).

## Supplementary Material

Crystal structure: contains datablocks I, global. DOI: 10.1107/S1600536809044079/fl2278sup1.cif

Structure factors: contains datablocks I. DOI: 10.1107/S1600536809044079/fl2278Isup2.hkl

Additional supplementary materials:  crystallographic information; 3D view; checkCIF report

## Figures and Tables

**Table 1:** Hydrogen-bond geometry (Å, °)

*D*—H⋯*A*	*D*—H	H⋯*A*	*D*⋯*A*	*D*—H⋯*A*
O6—H6⋯O3^i^	0.82	1.75	2.5712 (17)	177
O2—H2⋯O7^ii^	0.82	1.71	2.522 (2)	169
O8—H2*W*⋯O2^iii^	0.842 (9)	1.978 (10)	2.8199 (18)	179 (3)
O8—H1*W*⋯O4^i^	0.842 (9)	2.202 (13)	3.020 (2)	164 (3)
N1—H1*A*⋯O7^iii^	0.89	1.89	2.7788 (18)	177
N1—H1*B*⋯O5	0.89	2.31	3.0083 (19)	135
N1—H1*B*⋯O1^iv^	0.89	2.48	3.105 (2)	127
N1—H1*C*⋯O3^i^	0.89	1.94	2.821 (2)	168

Symmetry codes: (i) 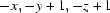; (ii) 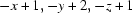; (iii) 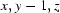; (iv) 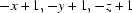.

**Fig. 1.**
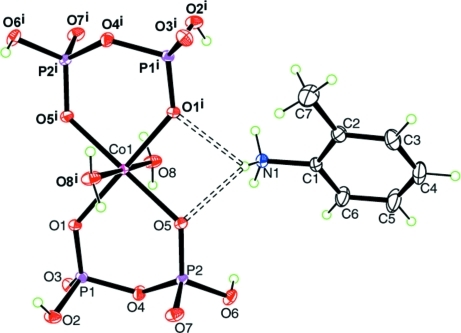
An ORTEP view of (I) with the atom-labelling scheme. Displacement ellipsoids are drawn at the 30% probability level. H atoms are represented as small spheres of arbitrary radii. Hydrogen bonds are represented as dashed lines.[Symmetry code: (i) 1 - x, 1 - y, 1 - z.]

**Fig. 2.**
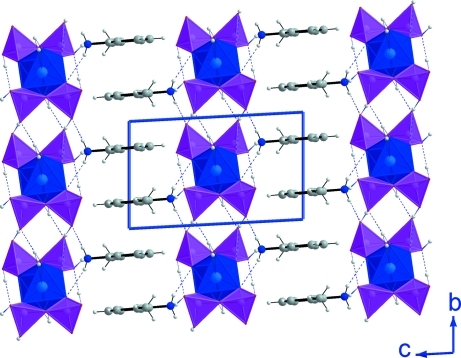
Projection of (I) along the a axis.

## supplementary crystallographic information

### Comment

Organic inorganic transition metal frameworks can be usefully employed in diverse areas, such as shape selective catalysis or adsorption (Cheetham *et al.*, 1999; Clearfield, 1998). In such compounds the transition metal plays a key role for building interesting topologies with one-, two- or three-dimensional networks. In these atomic arrangements, the transition element is coordinated generally to ligands *via* several donor atoms such as oxygen or nitrogen. In recent years, many researchers have focused on diphosphates because they are powerful ligands that can link metal ions through their oxygen atoms, and can play an essential role in the interaction between the metallic centers (Xu *et al.*, 2008).

The title compound, is built up from a diaquabis[dihydrogendiphosphato(2)]cobaltate(II) anion and two organic 2-methylanilinium cations (Fig. 1). A half of the complex anion and one organic cation constitute the asymmetric unit of (I).

The metal complex anions, interconnected *via* hydrogen bonds involving the two hydroxyl groups of H_2_P_2_O_7_^2-^ and the water molecule, develop a thick bi-dimensional layer of formula [Co(H_2_P_2_O_7_)_2_(H_2_O)_2_]^2n-^ perpendicular to the *c* axis (Fig. 2). The protonated organic cations 2-CH_3_C_6_H_4_NH_3_^+^ are anchored between these layers .

With regard to the inorganic arrangement, the Co atom is located on an inversion center and is surrounded by two symmetry related dihydrogendiphosphate ligands with a bent eclipsed conformation as seen by the P1—O4—P2 angle of 129.26 (7)% , and two water molecules in an octahedral coordination. Four external O atoms, OE, in the basal plane from two bidendate [H_2_P_2_O_7_] groups and the two remaining O atoms, OW, in the apical positions from the water molecule give a slightly distorted CoO_6_ octahedron. Within this octahedron, the Co—O distances range from 2.057 (1) to 2.149 (1) Å with Cu—OW distances longer than those of Co—OE. A similar coordination geometry around the central atom has also been observed in other *M*^II^O_6_ octahedra, *M*^II^ = Co or Ni, in organic diphosphate compounds (Essehli *et al.*, 2006; Gharbi *et al.*, 1994; Gharbi *et al.*, 2004).

Analysis of hydrogen bonds within (I), revealed an intricate network of O—H···O and N—H···O bonds which along with other interactions (electrostatic and Van der Waals) stabilize the whole structure. The O—H···O contacts, with O—H···O distances ranging from 2.522 (2) to 3.020 (2) Å, link the complex anions while the N—H···O bonds linking  the anions and cations are weaker since the N—H···O distances are longer, ranging from 2.779 (2) to 3.105 (2) Å. These H-bonds (Table 1) participate in the cohesion of the three-dimensional network (Fig 2).

### Experimental

Crystals of the title compound were prepared by adding an ethanol solution (10 ml) of 2-methylaniline (7.52 mmol) dropwise to a mixture of H_4_P_2_O_7_ (3.75 mmol) and CoCl_2_ (1.88 mmol) in water (20 ml). Good quality green prisms were obtained after a slow evaporation during few days at ambient temperature. The diphosphoric acid, H_4_P_2_O_7_, was produced from Na_4_P_2_O_7_ by using an ion-exchange resin (Amberlite IR 120).

### Crystal data

**Table:**

(C_7_H_10_N)_2_[Co(H_2_P_2_O_7_)_2_(H_2_O)_2_]	*Z* = 1
*M**_r_* = 663.19	*F*(000) = 341
Triclinic, *P*1	*D*_x_ = 1.754 Mg m^−^^3^
*a* = 7.440 (4) Å	Ag *K*α radiation, λ = 0.56085 Å
*b* = 7.455 (2) Å	Cell parameters from 25 reflections
*c* = 11.747 (3) Å	θ = 9–11°
α = 91.92 (3)°	µ = 0.53 mm^−^^1^
β = 94.09 (5)°	*T* = 298 K
γ = 104.67 (2)°	Prism, pink
*V* = 627.8 (4) Å^3^	0.33 × 0.26 × 0.23 mm

### Data collection

**Table:**

Enraf–Nonius CAD-4 diffractometer	*R*_int_ = 0.008
Radiation source: Enraf Nonius FR590	θ_max_ = 25.0°, θ_min_ = 2.2°
graphite	*h* = −11→11
Non–profiled ω scans	*k* = −11→11
4678 measured reflections	*l* = 0→17
4486 independent reflections	2 standard reflections every 120 min
3911 reflections with *I* > 2σ(*I*)	intensity decay: 7%

### Refinement

**Table:**

Refinement on *F*^2^	Primary atom site location: structure-invariant direct methods
Least-squares matrix: full	Secondary atom site location: difference Fourier map
*R*[*F*^2^ > 2σ(*F*^2^)] = 0.028	Hydrogen site location: inferred from neighbouring sites
*wR*(*F*^2^) = 0.078	H atoms treated by a mixture of independent and constrained refinement
*S* = 1.08	*w* = 1/[σ^2^(*F*_o_^2^) + (0.0409*P*)^2^ + 0.209*P*] where *P* = (*F*_o_^2^ + 2*F*_c_^2^)/3
4486 reflections	(Δ/σ)_max_ = 0.001
181 parameters	Δρ_max_ = 0.43 e Å^−^^3^
3 restraints	Δρ_min_ = −0.29 e Å^−^^3^

### Special details

**Table:**

Geometry. All e.s.d.'s (except the e.s.d. in the dihedral angle between two l.s. planes) are estimated using the full covariance matrix. The cell e.s.d.'s are taken into account individually in the estimation of e.s.d.'s in distances, angles and torsion angles; correlations between e.s.d.'s in cell parameters are only used when they are defined by crystal symmetry. An approximate (isotropic) treatment of cell e.s.d.'s is used for estimating e.s.d.'s involving l.s. planes.
Refinement. Refinement of *F*^2^ against ALL reflections. The weighted *R*-factor *wR* and goodness of fit *S* are based on *F*^2^, conventional *R*-factors *R* are based on *F*, with *F* set to zero for negative *F*^2^. The threshold expression of *F*^2^ > σ(*F*^2^) is used only for calculating *R*-factors(gt) *etc*. and is not relevant to the choice of reflections for refinement. *R*-factors based on *F*^2^ are statistically about twice as large as those based on *F*, and *R*- factors based on ALL data will be even larger.

### Fractional atomic coordinates and isotropic or equivalent isotropic displacement parameters (Å^2^)

**Table:**

	*x*	*y*	*z*	*U*_iso_*/*U*_eq_	
Co1	0.5000	0.5000	0.5000	0.01548 (6)	
P1	0.21765 (4)	0.73669 (4)	0.39318 (3)	0.01617 (7)	
P2	0.27309 (4)	0.74457 (4)	0.64170 (3)	0.01643 (7)	
O1	0.37007 (14)	0.64107 (14)	0.38682 (8)	0.02204 (18)	
O2	0.29044 (15)	0.94783 (14)	0.37491 (11)	0.0272 (2)	
H2	0.4017	0.9718	0.3647	0.041*	
O3	0.04241 (14)	0.65899 (15)	0.31845 (9)	0.0253 (2)	
O4	0.15097 (14)	0.73056 (16)	0.52080 (9)	0.02389 (19)	
O5	0.39173 (14)	0.61111 (14)	0.63563 (8)	0.02138 (18)	
O6	0.11785 (15)	0.68728 (15)	0.72493 (9)	0.0258 (2)	
H6	0.0630	0.5776	0.7116	0.039*	
O7	0.37403 (15)	0.94287 (14)	0.67018 (10)	0.0275 (2)	
O8	0.25633 (15)	0.27064 (15)	0.49415 (11)	0.0291 (2)	
N1	0.29299 (18)	0.25923 (18)	0.76160 (10)	0.0234 (2)	
H1A	0.3161	0.1554	0.7340	0.035*	
H1B	0.3821	0.3566	0.7445	0.035*	
H1C	0.1832	0.2687	0.7308	0.035*	
C1	0.2890 (2)	0.2541 (2)	0.88612 (12)	0.0242 (3)	
C2	0.4492 (2)	0.2523 (2)	0.95316 (14)	0.0313 (3)	
C3	0.4354 (3)	0.2471 (3)	1.07075 (16)	0.0469 (5)	
H3	0.5407	0.2471	1.1184	0.056*	
C4	0.2716 (4)	0.2419 (4)	1.11818 (16)	0.0550 (6)	
H4	0.2668	0.2390	1.1970	0.066*	
C5	0.1146 (3)	0.2411 (4)	1.04945 (18)	0.0579 (6)	
H5	0.0026	0.2356	1.0815	0.069*	
C6	0.1229 (3)	0.2486 (3)	0.93204 (15)	0.0439 (5)	
H6A	0.0173	0.2499	0.8849	0.053*	
C7	0.6297 (3)	0.2562 (4)	0.90335 (19)	0.0530 (6)	
H7A	0.6174	0.1429	0.8587	0.079*	
H7B	0.7264	0.2685	0.9639	0.079*	
H7C	0.6609	0.3596	0.8555	0.079*	
H2W	0.266 (3)	0.174 (2)	0.458 (2)	0.052 (7)*	
H1W	0.149 (2)	0.287 (4)	0.481 (2)	0.070 (9)*	

### Atomic displacement parameters (Å^2^)

**Table:**

	*U*^11^	*U*^22^	*U*^33^	*U*^12^	*U*^13^	*U*^23^
Co1	0.01533 (10)	0.01613 (11)	0.01672 (11)	0.00681 (8)	0.00249 (8)	0.00182 (8)
P1	0.01346 (13)	0.01654 (13)	0.01897 (14)	0.00477 (10)	0.00046 (10)	0.00225 (10)
P2	0.01503 (13)	0.01627 (13)	0.01831 (14)	0.00417 (10)	0.00368 (10)	−0.00060 (10)
O1	0.0232 (4)	0.0268 (5)	0.0211 (4)	0.0146 (4)	0.0039 (3)	0.0048 (3)
O2	0.0226 (5)	0.0166 (4)	0.0435 (6)	0.0046 (3)	0.0094 (4)	0.0045 (4)
O3	0.0192 (4)	0.0266 (5)	0.0276 (5)	0.0033 (4)	−0.0058 (4)	0.0029 (4)
O4	0.0175 (4)	0.0356 (5)	0.0208 (4)	0.0104 (4)	0.0031 (3)	0.0036 (4)
O5	0.0249 (4)	0.0240 (4)	0.0188 (4)	0.0124 (4)	0.0029 (3)	0.0005 (3)
O6	0.0244 (5)	0.0267 (5)	0.0252 (5)	0.0023 (4)	0.0113 (4)	−0.0023 (4)
O7	0.0236 (5)	0.0172 (4)	0.0402 (6)	0.0018 (4)	0.0081 (4)	−0.0043 (4)
O8	0.0194 (5)	0.0226 (5)	0.0449 (6)	0.0051 (4)	0.0037 (4)	−0.0048 (4)
N1	0.0260 (5)	0.0274 (6)	0.0187 (5)	0.0100 (4)	0.0022 (4)	0.0020 (4)
C1	0.0268 (6)	0.0295 (7)	0.0181 (5)	0.0105 (5)	0.0013 (5)	0.0029 (5)
C2	0.0302 (7)	0.0405 (8)	0.0249 (7)	0.0136 (6)	−0.0025 (5)	0.0005 (6)
C3	0.0520 (11)	0.0671 (14)	0.0254 (8)	0.0252 (10)	−0.0082 (7)	0.0032 (8)
C4	0.0703 (15)	0.0815 (17)	0.0207 (7)	0.0315 (13)	0.0095 (8)	0.0077 (9)
C5	0.0507 (12)	0.101 (2)	0.0299 (9)	0.0290 (13)	0.0178 (8)	0.0089 (11)
C6	0.0314 (8)	0.0799 (15)	0.0262 (7)	0.0233 (9)	0.0067 (6)	0.0074 (8)
C7	0.0297 (9)	0.0902 (18)	0.0426 (10)	0.0239 (10)	−0.0023 (8)	−0.0015 (11)

### Geometric parameters (Å, °)

**Table:**

Co1—O1	2.0574 (12)	N1—C1	1.4667 (18)
Co1—O1^i^	2.0574 (12)	N1—H1A	0.8900
Co1—O5	2.0752 (12)	N1—H1B	0.8900
Co1—O5^i^	2.0752 (12)	N1—H1C	0.8900
Co1—O8	2.1491 (14)	C1—C6	1.375 (2)
Co1—O8^i^	2.1491 (14)	C1—C2	1.384 (2)
P1—O1	1.4892 (11)	C2—C3	1.394 (2)
P1—O3	1.4919 (14)	C2—C7	1.496 (3)
P1—O2	1.5570 (11)	C3—C4	1.368 (3)
P1—O4	1.6108 (12)	C3—H3	0.9300
P2—O5	1.4909 (11)	C4—C5	1.371 (3)
P2—O7	1.4933 (12)	C4—H4	0.9300
P2—O6	1.5529 (13)	C5—C6	1.387 (3)
P2—O4	1.6160 (13)	C5—H5	0.9300
O2—H2	0.8200	C6—H6A	0.9300
O6—H6	0.8200	C7—H7A	0.9600
O8—H2W	0.842 (9)	C7—H7B	0.9600
O8—H1W	0.842 (9)	C7—H7C	0.9600
			
O1—Co1—O1^i^	180.00 (4)	Co1—O8—H1W	121 (2)
O1—Co1—O5	90.50 (5)	H2W—O8—H1W	111 (2)
O1^i^—Co1—O5	89.50 (5)	C1—N1—H1A	109.5
O1—Co1—O5^i^	89.50 (5)	C1—N1—H1B	109.5
O1^i^—Co1—O5^i^	90.50 (5)	H1A—N1—H1B	109.5
O5—Co1—O5^i^	180.00 (3)	C1—N1—H1C	109.5
O1—Co1—O8	91.82 (6)	H1A—N1—H1C	109.5
O1^i^—Co1—O8	88.18 (6)	H1B—N1—H1C	109.5
O5—Co1—O8	86.64 (6)	C6—C1—C2	122.21 (15)
O5^i^—Co1—O8	93.36 (6)	C6—C1—N1	118.02 (14)
O1—Co1—O8^i^	88.18 (6)	C2—C1—N1	119.77 (14)
O1^i^—Co1—O8^i^	91.82 (6)	C1—C2—C3	116.73 (17)
O5—Co1—O8^i^	93.36 (6)	C1—C2—C7	122.34 (15)
O5^i^—Co1—O8^i^	86.64 (6)	C3—C2—C7	120.94 (17)
O8—Co1—O8^i^	180.0	C4—C3—C2	122.00 (18)
O1—P1—O3	117.53 (7)	C4—C3—H3	119.0
O1—P1—O2	110.90 (7)	C2—C3—H3	119.0
O3—P1—O2	109.47 (7)	C3—C4—C5	119.95 (18)
O1—P1—O4	109.65 (7)	C3—C4—H4	120.0
O3—P1—O4	104.33 (7)	C5—C4—H4	120.0
O2—P1—O4	103.91 (7)	C4—C5—C6	119.9 (2)
O5—P2—O7	115.99 (7)	C4—C5—H5	120.1
O5—P2—O6	112.76 (7)	C6—C5—H5	120.1
O7—P2—O6	108.25 (7)	C1—C6—C5	119.22 (18)
O5—P2—O4	108.51 (6)	C1—C6—H6A	120.4
O7—P2—O4	108.93 (7)	C5—C6—H6A	120.4
O6—P2—O4	101.36 (7)	C2—C7—H7A	109.5
P1—O1—Co1	134.55 (7)	C2—C7—H7B	109.5
P1—O2—H2	109.5	H7A—C7—H7B	109.5
P1—O4—P2	129.26 (7)	C2—C7—H7C	109.5
P2—O5—Co1	132.40 (6)	H7A—C7—H7C	109.5
P2—O6—H6	109.5	H7B—C7—H7C	109.5
Co1—O8—H2W	114.2 (17)		

Symmetry codes: (i) −*x*+1, −*y*+1, −*z*+1.

### Hydrogen-bond geometry (Å, °)

**Table:**

*D*—H···*A*	*D*—H	H···*A*	*D*···*A*	*D*—H···*A*
O6—H6···O3^ii^	0.82	1.75	2.5712 (17)	177
O2—H2···O7^iii^	0.82	1.71	2.522 (2)	169
O8—H2W···O2^iv^	0.84 (1)	1.98 (1)	2.8199 (18)	179 (3)
O8—H1W···O4^ii^	0.84 (1)	2.20 (1)	3.020 (2)	164 (3)
N1—H1A···O7^iv^	0.89	1.89	2.7788 (18)	177
N1—H1B···O5	0.89	2.31	3.0083 (19)	135
N1—H1B···O1^i^	0.89	2.48	3.105 (2)	127
N1—H1C···O3^ii^	0.89	1.94	2.821 (2)	168

Symmetry codes: (ii) −*x*, −*y*+1, −*z*+1; (iii) −*x*+1, −*y*+2, −*z*+1; (iv) *x*, *y*−1, *z*; (i) −*x*+1, −*y*+1, −*z*+1.

## References

[bb1] Brandenburg, K. & Putz, H. (2005). *DIAMOND* Crystal Impact GbR, Bonn, Germany.

[bb2] Cheetham, A. K., Ferey, G. & Loiseau, T. (1999). *Angew. Chem. Int. Ed. Engl.* **38**, 3268–3292. 10602176

[bb3] Clearfield, A. (1998). *Chem. Mater.* **10**, 2801–2810.

[bb4] Enraf–Nonius (1994). *CAD-4 EXPRESS* Enraf–Nonius, Delft, The Netherlands.

[bb5] Essehli, R., El Bali, B., Lachkar, M., Svoboda, I. & Fuess, H. (2006). *Acta Cryst.* E**62**, m538–m541.

[bb6] Farrugia, L. J. (1997). *J. Appl. Cryst.* **30**, 565.

[bb7] Farrugia, L. J. (1999). *J. Appl. Cryst.* **32**, 837–838.

[bb8] Gharbi, A. & Jouini, A. (2004). *J. Chem. Crystallogr.* **34**, 11–13.

[bb9] Gharbi, A., Jouini, A., Averbuch-Pouchot, M. T. & Durif, A. (1994). *J. Solid State Chem.* **111**, 330–337.

[bb10] Harms, K. & Wocadlo, S. (1996). *XCAD4* University of Marburg, Germany.

[bb11] Sheldrick, G. M. (2008). *Acta Cryst.* A**64**, 112–122. 10.1107/S0108767307043930 18156677

[bb12] Xu, J. Y., Tian, J. L., Zhang, Q. W., Zhao, J., Yan, S. P. & Liao, D. Z. (2008). *Inorg. Chem. Commun.* **11**, 69–72.

